# The Monitoring and Cell Imaging of Fe^3+^ Using a Chromone-Based Fluorescence Probe

**DOI:** 10.3390/molecules29071504

**Published:** 2024-03-28

**Authors:** Yongjun Bian, Xingyu Qu, Fengying Zhang, Zhengwei Zhang, Jin Kang

**Affiliations:** 1 College of Chemistry and Chemical Engineering, Jinzhong University, Jinzhong 030619, China; quxy@jzxy.edu.cn (X.Q.); zhangzhw1225@163.com (Z.Z.); 18835118527@163.com (J.K.); 2 Department of Materials Science and Engineering, Jinzhong University, Jinzhong 030619, China

**Keywords:** chromone, fluorescent probe, Fe^3+^ ions, fluorescence quenching, cell imaging

## Abstract

A new structurally simple fluorescent **CP** probe based on chromone was designed and synthesized, and its structure was fully characterized using various analytical techniques. The **CP** probe displays a high selectivity and sensitivity for sensing Fe^3+^ with a “turn-off” fluorescence response over other metal ions in a DMSO/H_2_O (4:1, *v*/*v*) solution. The experiment results show that the **CP** probe is stable over a wide pH range of 2.0–12.0. The detection limit for Fe^3+^ was calculated to be 0.044 μmol•L^−1^. The molar ratio method indicated that the binding mode between the **CP** probe and Fe^3+^ is a 1:1 complex formation. HR-MS and density functional theory (DFT) calculations were also performed to further confirm the recognition mechanism. Both fluorescence imaging experiments and the MTT assay demonstrated that the **CP** probe was suitable for detecting intracellular Fe^3+^ and no significant cytotoxicity in living cells.

## 1. Introduction

Iron, as one of the essential microelements [1], is widespread in living organisms and plays crucial roles in a variety of fundamental biochemical processes, like oxygen metabolism, electron transfer, and enzyme catalysis [2,3,4]. In the human body, most irons are mainly stored in Fe^3+^ form [5]. When the Fe^3+^ content is within the proper level range, the body can function normally. Either the deficiency or the excess of Fe^3+^ could induce diseases such as hemochromatosis, heart failure, diabetes, and liver fibrosis [6,7,8,9]. Thus, it is very important to design a good method for the determination of Fe^3+^.

Compared with traditional detection methods, such as spectrophotometry, mass spectrometry, and atomic absorption spectroscopy [10,11,12], a method based on a fluorescent probe has attracted increasing interest due to its simple operation, high selectivity, and fast response. Currently, various fluorescent probes based on different fluorophores, such as naphthalimide, quinazolinone, rhodamine, and BODIPY, have been developed for the detection of Fe^3+^ [13,14,15,16,17,18]. Nevertheless, most of them were easily disrupted by other transition metal ions (e.g., Cu^2+^, Al^3+^, etc.), resulting in poor selectivity [19,20,21]. Therefore, it is still necessary to develop highly sensitive and selective Fe^3+^ fluorescent probes. Due to their excellent spectroscopic properties, many chromone-based derivatives have also been used as fluorescent probes. For example, Bai et al. proposed a fluorescent probe based on a chromone–dendron Schiff base to detect Mg^2+^ and Zn^2+^ [22]. Li et al. reported a chromone-derived probe for the monitoring of Al^3+^ based on a photoinduced electron-transfer mechanism [23]. A Cu^2+^ chromone derivative was also developed by Liu et al. by means of fluorescence quenching [24]. However, few chromone-based fluorescent probes were reported for the detection of Fe^3+^. Herein, we designed and synthesized a Schiff base fluorescent **CP** probe based on chromone with simple structure. The **CP** probe can be successfully applied to detect Fe^3+^ with high sensitivity and good selectivity. Meanwhile, fluorescence microscopy experiments demonstrated that the **CP** probe can also be used to monitor Fe^3+^ in living cells.

## 2. Results and Discussion

**Scheme 1:**
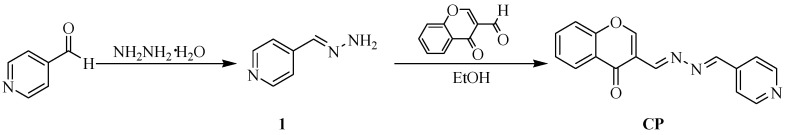
Synthesis route for the **CP** probe.

The general procedure for the synthesis of the **CP** probe is shown in Scheme 1. Intermediate compound **1** was prepared according to the reported method [25]. The **CP** probe was fully characterized by ^1^H NMR, ^13^C NMR, IR, and HR-MS.

### 2.1. Characterization of the **CP** Probe

**Figure 1:**
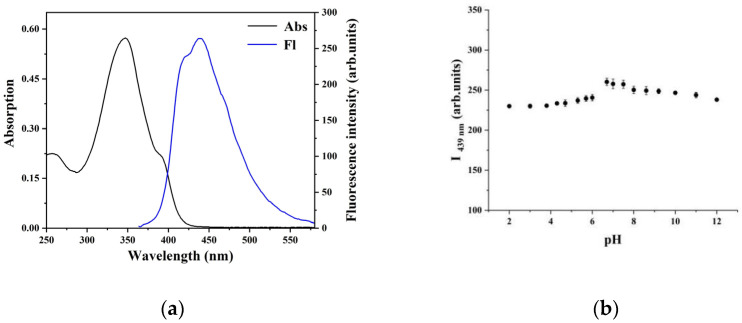
(**a**) The UV/visible absorption and emission spectra of the **CP** probe (10 μmol•L^−1^) in DMSO/H_2_O mixed solvent (4:1, *v*/*v*). (**b**) Effect of pH on the fluorescence intensity of the **CP** probe (10 μmol•L^−1^) in DMSO/H_2_O mixed solvent (4:1, *v*/*v*) at 439 nm.

The UV/visible absorption and emission spectra of the **CP** probe in DMSO/H_2_O mixed solvent is investigated, as shown in Figure 1a. The absorption maximum of **CP** probe is centered at approximately 345 nm due to molecular skeleton group π→π* transition. Exciting at 345 nm in the **CP** probe provides an emission band at 439 nm. Meanwhile, we also studied the effect of pH on the fluorescence of the **CP** probe (Figure 1b). In the pH range of 2.0–12.0, there was no significant change in fluorescence intensity, which demonstrated that it is insusceptible to the change of acid–base solution.

### 2.2. Fluorescence Detection and Selectivity of the **CP** Probe with Fe^3+^

**Figure 2:**
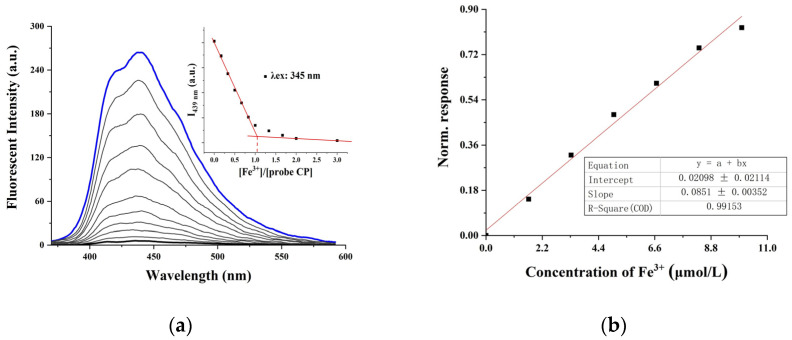
(**a**) Fluorescence spectra of the **CP** probe (10 μmol•L^−1^) upon addition of Fe^3+^ in mixed DMSO/H_2_O (4:1, *v*/*v*) solution (λex = 345 nm). (Inset) The emission intensity at 439 nm of **CP** probe changes as a function of the ratio of Fe^3+^ concentration to **CP** probe concentration. (**b**) Dependence of normalized signal on concentration of Fe ^3+^ ions.

Next, we conducted spectrophotometric titration experiments to investigate the response of the **CP** probe on Fe^3+^ ions in a DMSO/H_2_O mixed solvent, and the results are shown in Figure 2. The emission properties of the **CP** probe were explored using the gradual addition of Fe^3+^ (0 to 3 equiv.) by exciting the solution at 345 nm. As shown in Figure 2a, upon the gradual addition of Fe^3+^ to the **CP** solution, a decrease in fluorescence intensity at 439 nm can be observed with a virtually unchanged peak position; the intensity linearly decreased and remained unchanged after one equivalent addition of Fe^3+^. A plot of the intensity of the **CP** probe at 439 nm versus [Fe^3+^] initially showed linear behavior and then remained constant, which prompted us to propose a 1:1 binding between the **CP** probe and Fe^3+^ (Figure 2a Inset). The fluorescence response of **CP** toward Fe^3+^ is normalized as R = 1 − Ii/Imax [26], where Imax is the fluorescence response of **CP** without Fe^3+^, Ii is the luminescent intensity of **CP** with Fe^3+^. The lowest detection limit was 0.044 μmol•L^−1^. A good linear relationship between the fluorescence response and concentration of Fe^3+^ was given with a 0.9915 correlation coefficient (Figure 2b).

**Figure 3:**
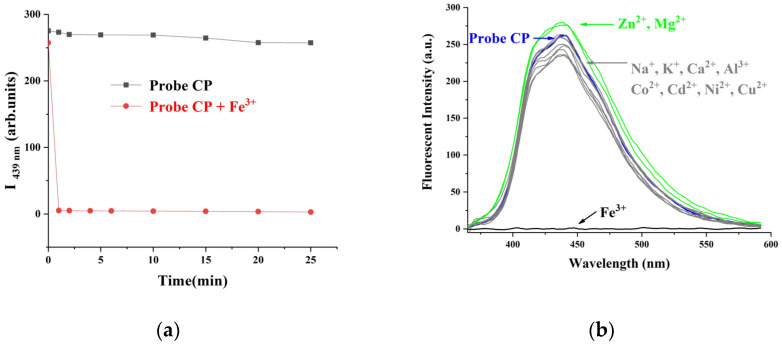
(**a**) Fluorescence intensity of the **CP** probe (10 μmol•L^−1^) at 439 nm before and after adding Fe^3+^ (0.03 mmol•L^−1^) over time. (**b**) Fluorescence spectra of the **CP** probe (10 μmol•L^−1^) upon addition of 1 mmol•L^−1^ different metal ions (Na^+^, K^+^, Ca^2+^, Mg^2+^, Zn^2+^, Ni^2+^, Co^2+^, Cu^2+^, Cd^2+^, and Al^3+^) and 0.03 mmol•L^−1^ Fe^3+^ in DMSO/H_2_O (4:1, *v*/*v*) mixed solution.

The specificity of the **CP** probe toward Fe^3+^ was also determined, and the results are shown in Figure 3. First, the effect of response time on the fluorescence emission intensity of the **CP** probe at 439 nm with and without Fe^3+^ was studied. When Fe^3+^ was added, the fluorescence emission intensity at 439 nm completely decreased in 1 min and became saturated for 25 min, as shown in Figure 3a. Thus, the results show that this **CP** probe could be applied for the real-time detection of Fe^3+^.

Subsequently, we investigated the fluorescence response of **CP** to all sorts of common metal ions, such as Na^+^, K^+^, Ca^2+^, Mg^2+^, Zn^2+^, Ni^2+^, Co^2+^, Cu^2+^, Cd^2+^, Fe^3+^, and Al^3+^, in order to evaluate selectivity, which is a very important parameter for a fluorescence probe; the results are shown in Figure 3b. When these ions were separately added to **CP** in a DMSO/H_2_O (4:1, *v*/*v*) mixed solution, only Fe^3+^ completely quenched the fluorescence of **CP**. Other metal ions only induced slight changes (either fluorescence enhancement or quenching) in the selected working conditions, even if their concentration was 33 times greater than that of Fe^3+^ ions. Some other ions, including anions and ROS, such as SO_4_^2−^, HSO_3_^−^, S_2_O_3_^2−^, SCN^−^, C_2_O_4_^2−^, NO_2_^−^, Cl^−^, Br^−^, I^−^, H_2_O_2_, and OH·, were also studied to investigate the fluorescence response of the **CP** probe. The results show that additions of these other ions did not cause any discernible changes (Figure S1, Supplementary Information). These observations showed that the **CP** probe had good selectivity for Fe^3+^ ions and could be used to effectively detect them.

### 2.3. Sensing Mechanism of the **CP** Probe with Fe^3+^

**Figure 4:**
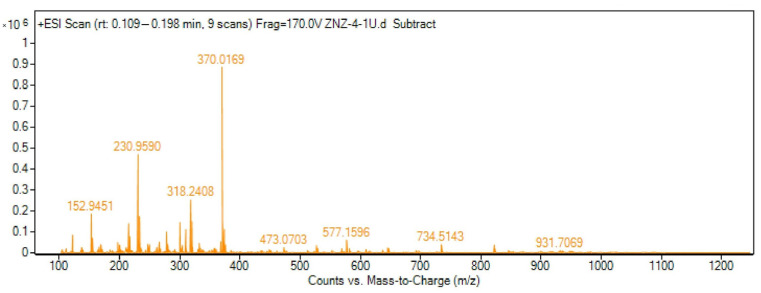
MS spectrum of **CP**-Fe^3+^-2H_2_O complex.

The molar ratio method proved that the **CP** probe and Fe^3+^ formed a 1:1 stoichiometry complex, as shown in the inset to Figure 2 [27,28,29,30]. To further prove the sensing mechanism of the **CP** probe toward Fe^3+^, ESI-MS spectrometry analysis and a density functional theory (DFT) calculation were applied. Subsequently, the ESI-MS spectrometry of the forming complex **CP**-Fe^3+^ was first determined, in which the peak at *m*/*z* = 370.0169 was assigned to [**CP** + Fe^3+^ + 2H_2_O + H^+^], supporting the 1:1 binding mode of the **CP** probe and Fe^3+^ (Figure 4). The IR spectra also indicated that the **CP** probe and Fe^3+^ formed a coordination compound, where the characteristic absorption moieties of C=O- and C=N-situated chromones at 1641 cm^−1^ and 1615 cm^−1^ shifted to 1616 cm^−1^ and 1553 cm^−1^, respectively (Figure S2, Supplementary Information).

**Figure 5:**
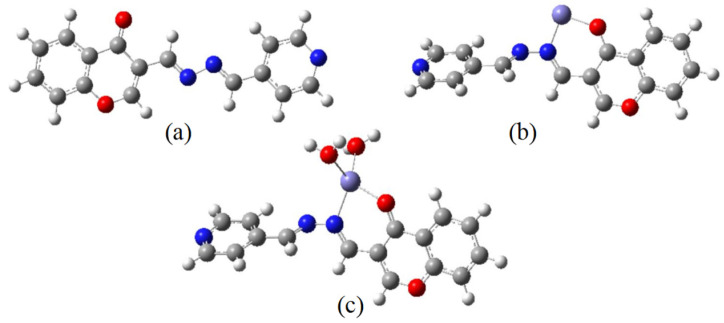
Energy-optimized structures of the **CP** probe (**a**), **CP**-Fe^3+^ (**b**), and **CP**-Fe^3+^-2H_2_O complex (**c**).

A DFT calculation was carried out using Gaussian 09 [31] software with the help of the GaussianView 5.0 visualization program. The structures of the probe and the complexes were fully optimized using the B3LYP/6-311++G(d,p) basis set and Lanl2dz basis set in a DMSO/H_2_O mixed solution according to the experimental procedure, respectively. For this, we performed an optimization using the polarizable continuum model (PCM) method. Figure 5 shows the energy-optimized structures of the probe and its complexes. Figure 5a indicated that the structure of **CP** probe is planar. Because of the repulsive force between two hydrogen atoms of imine moiety, the structures of the complexes are nonplanar (Figure 5b,c). Additionally, the total energy values of the **CP** probe and the complex **CP**-Fe^3+^ (Figure 5b) were −931.2326582 a.u. and −2194.3728797 a.u., respectively. Again, the total energy of the complex **CP**-Fe^3+^-2H_2_O (Figure 5c) was lower (−2347.3856608 a.u.) than that of both **CP** and **CP**-Fe^3+^, which implied a higher stability. This result was in accordance with that of ESI-MS spectrometry analysis.

**Figure 6:**
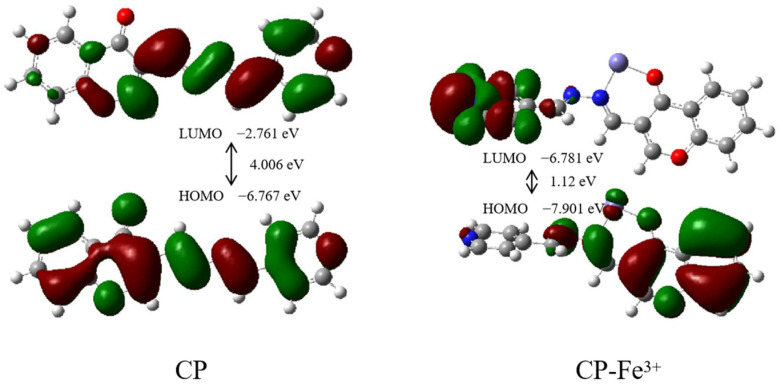
Energy level diagram of **CP** probe and the **CP**-Fe^3+^ complex for the HOMO and LUMO.

**Figure 7:**
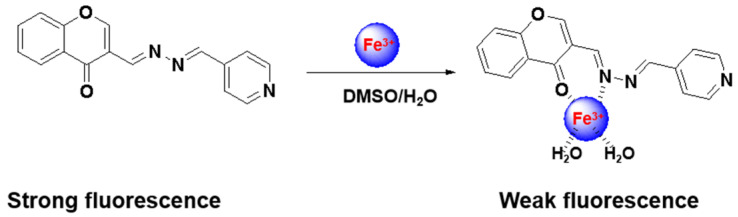
Proposed recognition mechanism for the **CP** probe.

Furthermore, the energy of highest occupied molecular orbital (HOMO) and the lowest unoccupied molecular orbital (LUMO) of the **CP** probe and its complex **CP**-Fe^3+^, along with their spatial distribution, was calculated, as shown in Figure 6. For the **CP** probe, both HOMO and LUMO essentially resided on its entire skeleton. In the case of the complex **CP**-Fe^3+^, the HOMO was mainly distributed in the chromone moiety, but the LUMO was mostly situated on the pyridine moiety. It can be seen in the frontier molecular orbital diagram that the HOMO of the complex **CP**-Fe^3+^ did not overlap with its LUMO. After the binding of Fe^3+^ to **CP**, the HOMO–LOMO energy gap (ΔΕ) was lowered from 4.01 eV to 1.12 eV, which further stabilized the **CP**-Fe^3+^ system. Hence, the electron transfer from the chromone moiety to the pyridine moiety easily occurred, resulting in fluorescence quenching. The probable detection mechanism for monitoring Fe^3+^ is described in Figure 7.

### 2.4. Practical Application of **CP** Probe

**Figure 8:**
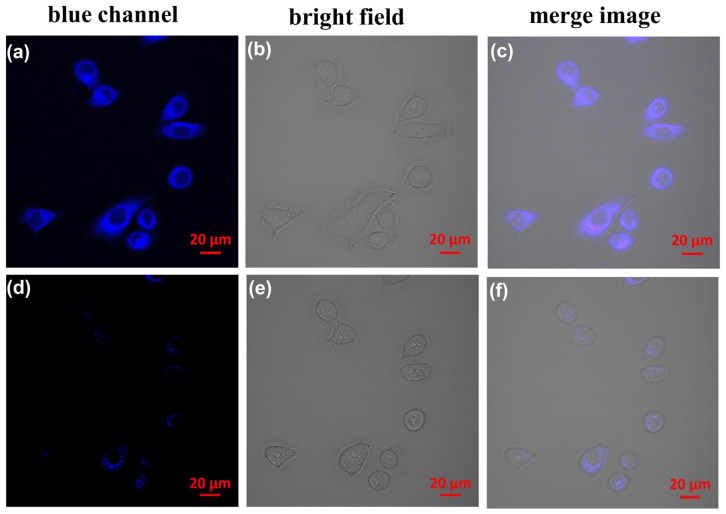
Confocal fluorescence, bright field images, and overlay image of the **CP** probe in HeLa cells. (**a**) Cells incubated with 5 μmol•L^−1^ of **CP** probe for 10 min at 37 °C upon excitation at 405 nm. (**d**) Cells supplemented with 50 μmol•L^−1^ of Fe^3+^ in growth media for 10 min at 37 °C upon excitation at 405 nm. (**b**,**e**) Bright field image of cells shown in the panel. (**c**,**f**) Overlay image of cells is shown in panel.

Next, to evaluate its practical application, the **CP** probe was subsequently used for living cell fluorescence imaging to detect Fe^3+^ ions. As shown in Figure 8, when the HeLa cells were incubated for 10 min at 37 °C with a 5 μmol•L^−1^ **CP** probe, a significant blue emission from the intracellular region was observed upon excitation at 405 nm (Figure 8a). After the cells were treated with 50 μmol•L^−1^ Fe^3+^ in the growth medium for 10 min at 37 °C, only weak intracellular fluorescence was observed in the microscope images (Figure 8d). Both bright field (Figure 8b,e) and overlay measurements (Figure 8c,f) with the Fe^3+^ and **CP** probe after treatment confirmed that the cells were viable for imaging experiments. The results confirm that the **CP** probe can monitor Fe^3+^ ions in a biological environment.

The cell viability of the probe is also an important parameter for its practical application. Therefore, the MTT assay experiments were conducted to evaluate the cell viability of the **CP** probe using three different kinds of cells, including HeLa, HepG2, and MCF-7 (Figures S3 and S4, Supplementary Information). Cell viability was monitored for 24 h after treatment with the **CP** probe over a wide range of concentrations (0–50 μmol•L^−1^). The results demonstrate that the **CP** probe did not negatively affect cell viability up to concentrations 20 μmol•L^−1^ for HeLa cells or 50 μmol•L^−1^ for HepG2 and MCF-7 cells, which showed that this probe had no prominent cytotoxicity to cells. Therefore, this **CP** probe could be an excellent device that has great potential for detecting Fe^3+^ in living cells.

## 3. Materials and Methods

### 3.1. Materials and Instruments

Both pyridine-4-carboxaldehyde and chromone-3-carboxaldehyde were purchased from HEOWNS Biochem Technologies LLC, Tianjin, China. Unless otherwise noted, all other reagents and solvents were commercially available and used without further purification. NMR spectra were recorded using a Bruker Avance NEO 600 MHz spectrometer (at 600 MHz for ^1^H NMR or 150 MHz for ^13^C NMR; Rheinstetten, Germany). A WQF-510A FT-IR spectrometer (Beijing Rayleigh Analytical Instrument Co., Ltd., Beijing, China) recorded the infrared spectroscopy in KBr discs in the 400–4000 cm^−1^ region. The melting points were measured on a Beijing Cossim X-5T micro-melting point apparatus (Beijing Century Letter Scientific instrument Co., Ltd., Beijing, China). HRMS (high-resolution mass spectra) was performed using an Aglient 7250 spectrometer (Santa Clara, CA, USA). Absorption spectra and fluorescence spectra were obtained using a TU-1901 UV-Vis spectrophotometer (Beijing Puxi General Instrument Co., Ltd., Beijng, China) and a Varian Cary Elipses spectro fluorophotometer (Palo Alto, CA, USA), respectively.

### 3.2. Synthesis of the **CP** Probe

A solution of chromone-3-carboxaldehyde in EtOH (5 mL) was added dropwise to a suspension of compound **2** (0.21 g, 1.72 mmol) EtOH (10 mL) over 5 min in an ice bath and the mixture was continually stirred for 8 h. Then, the precipitate was filtered and washed with EtOH. Finally, the solid was recrystallized from EtOAc to afford the **CP** probe (0.30 g, 66%) as a white solid. R_f_ = 0.34 (V_(EtOAc)_:V_(Petroleum ether)_ = 2:1), m.p. 220.5–221.3 °C. ^1^H NMR (CDCl_3_, 600 MHz), δ: 8.97 (s, 1H), 8.81 (s, 1H), 8.78 (d, *J* = 6 Hz, 2H), 8.56 (s, 1H), 8.33 (d, *J* = 12 Hz, 1H), 7.80–7.75 (m, 3H), 7.57 (d, *J* = 6 Hz, 1H), 7.52 (t, *J* = 6 Hz, 1H); ^13^C NMR(CDCl_3_, 150 MHz), δ: 175.60, 159.39, 156.25, 156.06, 155.73, 150.52, 140.96, 134.34, 126.21, 126.15, 124.26, 122.11, 118.45; IR(KBr)ν, cm^−1^: 3049, 1641, 1562, 1347, 1230, 991, 761, 661, 536; HR-MS(ESI): *m*/*z* calcd. for C_16_H_11_N_3_O_2_Na [M + Na^+^]: 300.0743, found: 300.0744.

### 3.3. Procedures for Metal Ions Sensing

Stock solutions of various metal ions (0.1 mol•L^−1^), such as Na^+^, K^+^, Ca^2+^, Mg^2+^, Zn^2+^, Ni^2+^, Co^2+^, Cu^2+^, Cd^2+^, and Al^3+^, were prepared in deionized water. Fe^3+^ (0.001 mol•L^−1^) is prepared in deionized water. For titration, the **CP** probe (3 mL, 10 μmol•L^−1^, dimethyl sulfoxide–water mixed solution) was added to a quartz optical cell of 1 cm optical path length. Metal ion stock solution was gradually added to the **CP** probe through a micro-pipette. The test samples for the evaluation of selectivity were obtained by adding appropriate amounts of metal ion stock solution to the **CP** probe (3 mL, 10μmol•L^−1^, dimethyl sulfoxide-water mixed solution). Fluorescence intensity was recorded at an excitation of 345 nm within 350–600 nm.

### 3.4. DFT Calculation

DFT calculation was performed in a binary solvent (DMSO/H_2_O, *v*/*v*, 4/1) using Gaussian 09. The calculation method is as follows. The static dielectric constant (EPS) and dynamic dielectric constant (EpsInf) of DMSO and H_2_O were calculated. The EPS and EpsInf values of DMSO were 46.8260 and 2.0079, respectively. The EPS and EpsInf values of H_2_O were 78.3553 and 1.7778, respectively. Then, according to the volume ratio, the static dielectric constant and dynamic dielectric constant of the mixed solvent with DMSO and H_2_O equal to 4:1 were determined. In the energy-optimized structure of probes and complexes, we used “scrf = (PCM, solvent = generic, read)” keywords.

### 3.5. Cell Culture and Fluorescence Bioimaging

The HeLa cell line was provided by Chemistry and Chemical Engineering of Shanxi University (Shanxi, China). The MTT assays for HepG2 and MCF-7 cells were conducted by Shiyanjia Lab (www.Shiyanjia.com, 21 February 2024). The cells were cultured in DMEM (4.5 g of glucose/L) supplemented with 10% FBS at 37 °C and 5% CO_2_, seeded into 96-well plates, and then stored for 12 h. The cells were washed with PBS buffer before use and then incubated with a 5 µmol•L^−1^ **CP** probe in a PBS buffer for another 10 min at 37 °C. The experiments were also carried out over 10 min in the same medium supplemented with 50 μmol•L^−1^ FeCl_3_·6H_2_O in order to assess Fe^3+^ uptake. Cell imaging was then carried out after washing the cells with PBS buffer 3 times. Confocal fluorescence imaging was performed with an Olympus FluoView FV1000 confocal microscope (Tokyo, Japan). Cells incubated with the **CP** probe were excited at 405 nm using a multi-line argon laser.

## 4. Conclusions

In summary, a new fluorescent **CP** probe was synthesized and structurally characterized. The recognition properties for Fe^3+^ were also evaluated. It displayed a high sensitivity and selectivity toward Fe^3+^, no significant optical interference arose from the other examined metal ions such as Na^+^, K^+^, Ca^2+^, Mg^2+^, Zn^2+^, Ni^2+^, Co^2+^, Cu^2+^, Cd^2+^ and Al^3+^. **CP** probe exhibited a fast fluorescence response within 1 min and a low detection limit of 0.044 μmol•L^−1^ for Fe^3+^. The stoichiometry ratio of **CP** probe binding with Fe^3+^ was verified as 1:1. The recognition mechanism of fluorescence quenching was the photoinduced electron transfer (PET) process between the **CP** probe and Fe^3+^ and the destruction of the large π-system of the **CP** probe. Further investigation demonstrated that the **CP** probe can be applied for the bioimaging of Fe^3+^ in living cells.

## Data Availability

Data are contained within the article and Supplementary Information.

## Supplementary Materials

The following supporting information can be downloaded at: https://www.mdpi.com/article/10.3390/molecules29071504/s1, Figure S1: Fluorescence spectra of **CP** probe for other species including anions and ROS; Figure S2: IR spectra for mechanism; Figure S3: MTT assay of **CP** probe at 24 h; Figure S4: MTT assay of **CP** probe for HepG2 and MCF-7 cells at 24 h; Figure S5: ^1^H NMR spectrum of **CP**; Figure S6: ^13^C NMR spectrum of **CP**; Figure S7: HR-MS spectrum of **CP**.
