# Supplementary material for: The Monitoring and Cell Imaging of Fe3+ Using a Chromone-Based Fluorescence Probe
Source: Molecules. 2024 Mar 28;29(7):1504. doi: 10.3390/molecules29071504 (PMC11013790; doi:10.3390/molecules29071504)
Supplement: Supplementary file 1 [file molecules-29-01504-s001.zip › molecules-2865816-supplementary.pdf]

Molecules

Supporting Information

Monitoring and Cell Imaging of Fe^{3+} Using a Chromone-based Fluorescence Probe

Yong-jun Bian [1,*], Xing-yu Qu [1], Feng-ying Zhang [2,*], Zheng-wei Zhang [1] and Jin Kang [1]

[1] College of Chemistry and Chemical Engineering, Jinzhong University, Jinzhong, P.R. China；yjbian2013@jzxy.edu.cn(Y.-J.B.); quxy@jzxy.edu.cn(X.-Y.Q.); zhangzhw1225@163.com (Z.-W.Z.); 18835118527@163.com(J.K.)

[2] Department of Materials Science and Engineering, Jinzhong University, Jinzhong 030619, China; zfy13233005848@163.com(F.-Y.Z.)

***** Correspondence: yjbian2013@jzxy.edu.cn(Y.-J.B.); zfy13233005848@163.com(F.-Y.Z.)

目　录

Figure S1. Fluorescence spectra of probe **CP** for other species including anions and ROS……………………………………S2

Figure S2 IR spectra for mechanism………………………………………………………………………………………….S2

Figure S3. MTT assay of probe **CP** at 24 h……………………………………………………………………………………...S3

Figure S4 MTT assay of probe **CP** for HepG2 and MCF-7 cells at 24 h……………………………………………………….S3

Figure S5. ^{1}H NMR spectrum of probe **CP**……………………………………………………………………………………..S4

Figure S6. ^{13}C NMR spectrum of probe **CP**………………………………………………………………………………..S4

Figure S7. HR-MS spectrum of probe **CP**…………………………………………………………………………………….S5

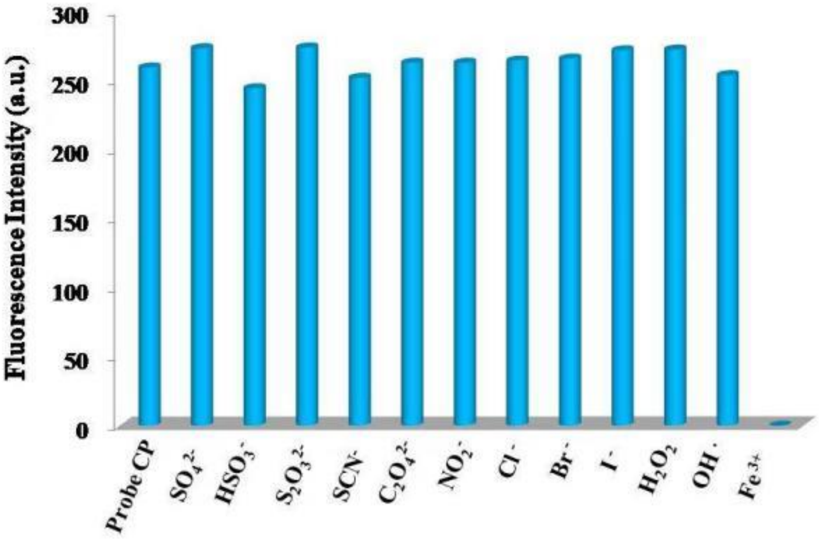

Figure S1 Fluorescence spectra of probe **CP** (10 μmol•L^{-1}) upon addition of 1 mmol•L^{-1} different anions and ROS (SO_4^{2-}, HSO_3^-, $S_2O_3^{2-}$, SCN^-, $C_2O_4^{2-}$, NO_2^-, Cl^-, Br^-, I^-, H_2O_2 , OH ·) and 0.03 mmol•L^{-1} Fe^{3+} in DMSO / H_2O (4:1, v/v) mixed solution.

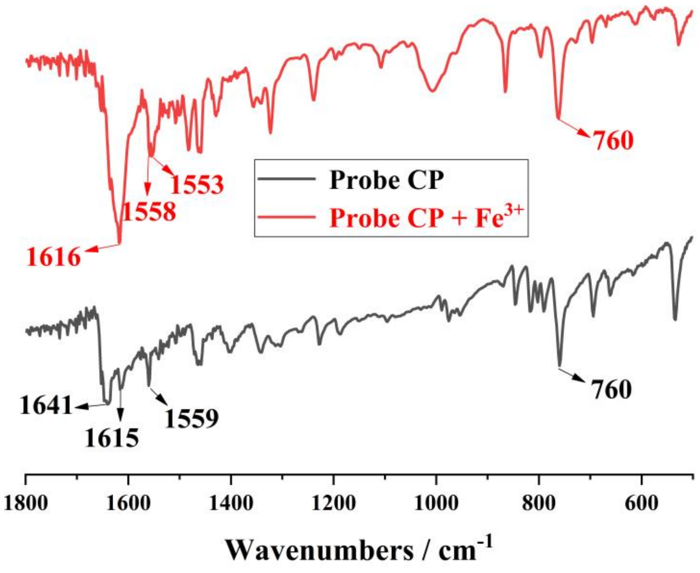

Figure S2 IR spectra of probe CP (black) and CP+Fe^{3+} complex (red)

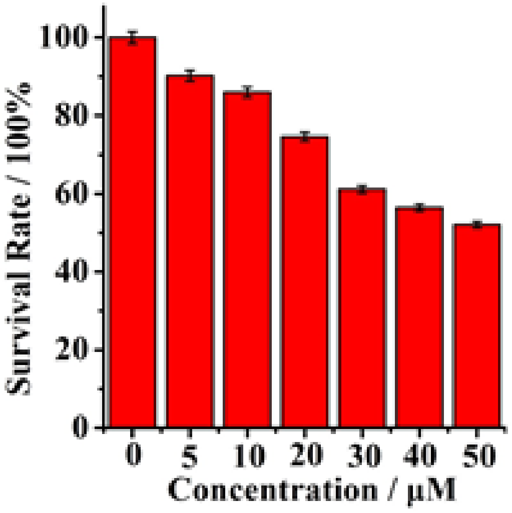

Figure S3 MTT assay of probe **CP** for HeLa cells at 24 h

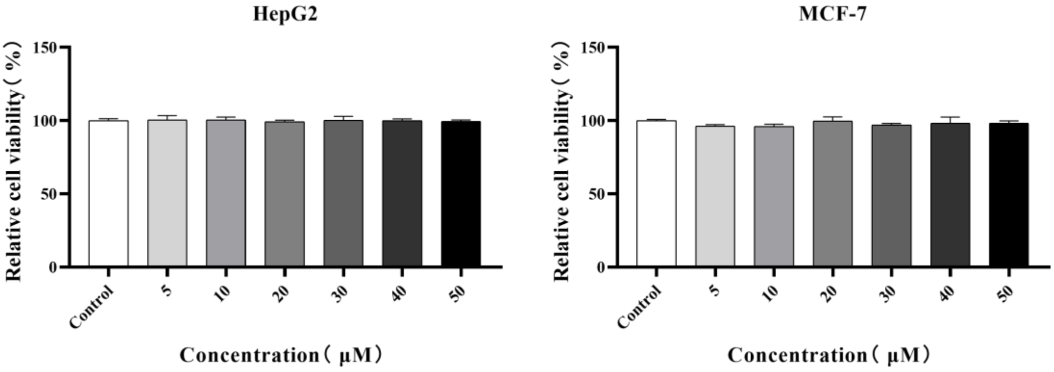

Figure S4 MTT assay of probe **CP** for HepG2 and MCF-7 cells at 24 h

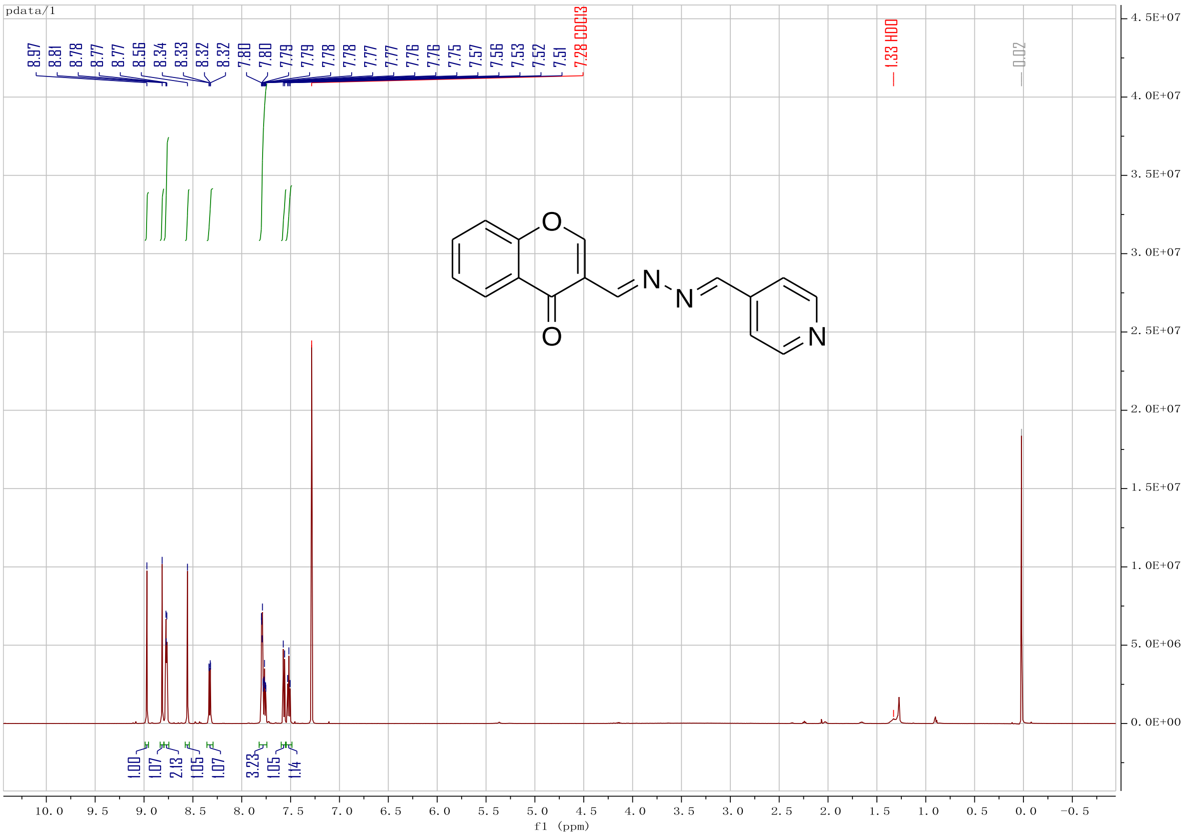

Figure S5 ¹H NMR spectrum of probe **CP** in $CDCl_3$.

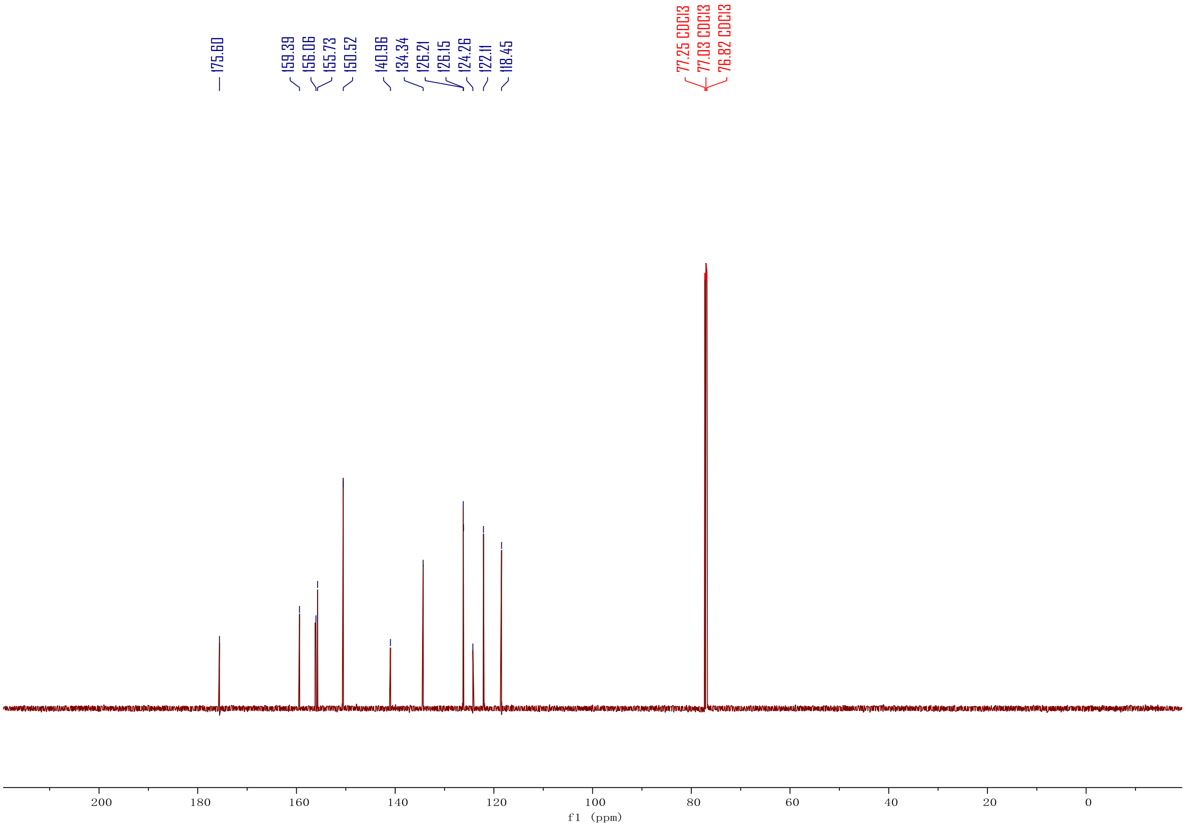

Figure S6 ¹³C NMR spectrum of probe **CP** in $CDCl_3$

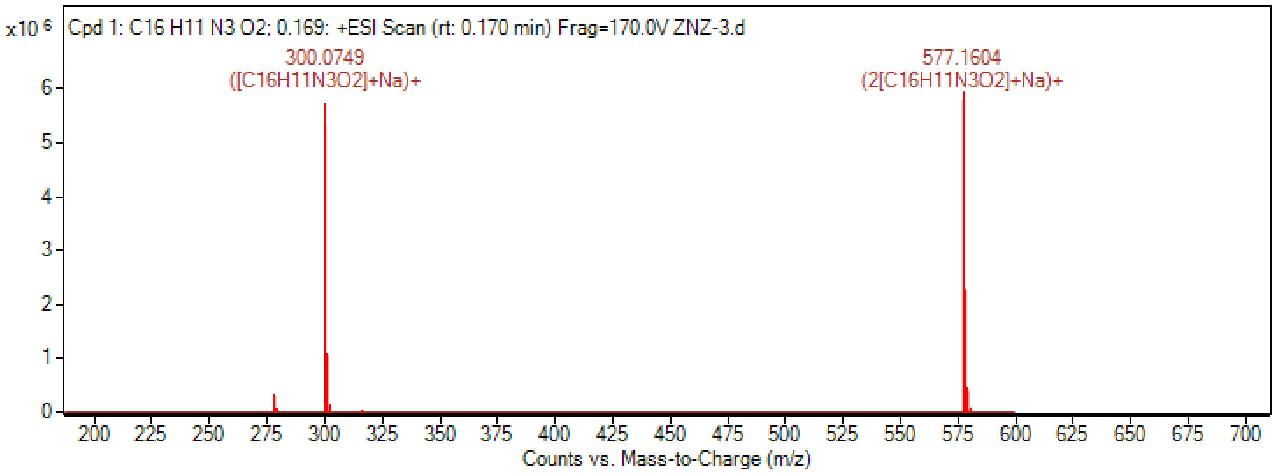

Figure S7 HR-MS spectrum of probe **CP**
